# Application of Acupuncture in Diabetic Vascular Complications: Efficacy and Potential Mechanisms

**DOI:** 10.1155/jdr/6934081

**Published:** 2025-09-22

**Authors:** Zhifeng Wang, Ying Chen, Ting Shi, Jiao Yang, Daman Tian, Hongyan Wu, Rong Zhao, Ruirui Wang, Liwei Xing

**Affiliations:** ^1^First Clinical Medical College, Yunnan University of Chinese Medicine, Kunming, Yunnan, China; ^2^Second Clinical Medical College, Yunnan University of Chinese Medicine, Kunming, Yunnan, China; ^3^College of Chinese Materia Medica, Yunnan University of Chinese Medicine, Kunming, Yunnan, China

**Keywords:** acupuncture, diabetic complications, integration and innovation, macrovascular complications, microvascular complications

## Abstract

Diabetes and its complications are a major global public health issue. Conventional Western medicine has limitations in efficacy and safety in managing complications, while acupuncture, with multitarget regulation and low side effects, serves as an important supplementary therapy. This review discusses how acupuncture relieves macrovascular complications, such as angina and cerebral reperfusion, by inhibiting NF-*κ*B, improving vascular endothelial function, and regulating autonomic nerves. In addition, acupuncture delays microvascular complications, such as retinopathy and proteinuria via vasodilation, anti-inflammatory effects, antioxidative effects, and neurotrophic promotion. Acupuncture also benefits other complications by enhancing microcirculation and neuroendocrine function. The mechanisms of acupuncture involve regulating the metabolic-inflammatory-neurovascular network, activating pathways (such as GLP-1 and SDF-1), and repairing cellular structures. Modern technologies—including artificial intelligence (AI) for individualized acupoint selection, functional magnetic resonance imaging (fMRI) for central regulation visualization, and biomaterial combination for diabetic foot repair—enhance the precision of acupuncture. However, clinical translation of acupuncture faces the following challenges: fragmented mechanisms; insufficient clinical evidence, including small samples, short follow-ups, and a lack of long-term safety data when used with new hypoglycemics; and technical nonstandardization, such as inconsistent acupoint selection, nonuniform operation parameters, and poor adaptability of AI to traditional Chinese medicine syndrome differentiation. Future research should deepen the exploration of the neuroendocrine–immune network via interdisciplinary integration, conducting large-sample long-term trials, establishing standardized protocols, and validating AI/fMRI-assisted precision acupoint selection to accelerate the transition of acupuncture from adjuvant to precision therapy to improve patients' quality of life.

## 1. Introduction

According to the data from the 2025 Global Diabetes Map released by the International Diabetes Federation (IDF), the number of adults aged 20–79 with diabetes worldwide has reached 589 million [1]. Diabetic complications, encompassing macrovascular complications (such as cardiovascular and cerebrovascular diseases) and microvascular complications (such as chronic kidney disease [CKD], retinopathy, and peripheral neuropathy), are the leading causes of disability and mortality among patients; approximately 40% of diabetic patients have comorbid cardiovascular diseases, one-third of diabetic patients suffer from CKD, and nearly 25% of diabetic patients develop retinopathy [2, 3]. Medical expenditures attributed to diabetic complications account for over 60% of total healthcare costs, and the incidence of complications has increased significantly since the COVID-19 pandemic in 2019 [4], further exacerbating the disease burden.

Although conventional Western medicine (including certain novel agents, such as sodium–glucose cotransporter 2 [SGLT2] inhibitors and glucagon-like peptide-1 [GLP-1] receptor agonists) can effectively control blood glucose, limitations remain in the prevention and treatment of both macrovascular and microvascular complications. A 2024 multicenter study has demonstrated that while combination therapy with these novel drugs reduces the short-term risks of cardiovascular events and end-stage renal disease, the long-term efficacy in intervening with macrovascular lesions, such as the progression of atherosclerotic plaques, remains unclear. Additionally, evidence supporting the effects of these novel drugs on microvascular complications, such as the reversal of retinopathy or the inhibition of renal fibrosis, is still insufficient, and these drugs have safety concerns, such as gastrointestinal reactions and ketosis [5]. A 2023 network meta-analysis has revealed that even with optimized drug regimens, the mortality rates of severe cardiovascular events and end-stage renal disease remain high and have not significantly decreased compared to traditional therapies [6]. These limitations result in persistently high incidence of complications and mortality rates due to complications, imposing tremendous physical, mental, and economic burdens on patients.

In recent years, the unique advantages of acupuncture in treating macrovascular and microvascular complications of diabetes have gradually emerged. For macrovascular lesions, acupuncture delays the progression of atherosclerosis by improving vascular endothelial function and inhibiting oxidative stress and inflammatory responses [7]. In terms of microvascular complications, acupuncture regulates the insulin signaling pathway and promotes the protection and regeneration of pancreatic islet *β*-cells to address the core issue of abnormal blood glucose regulation, and it also repairs peripheral nerves by improving the conduction velocity of peripheral nerves, thereby enhancing the clinical efficacy in treating diabetic peripheral neuropathy (DPN) [8, 9]. In clinical practice, when used alone or in combination with Western medicine, acupuncture results in a more significant reduction in the risk of complication progression compared to pure Western medicine treatment, with fewer side effects [10].

With the development of modern acupuncture techniques, such as electroacupuncture (EA), warm acupuncture, and auricular acupuncture, combined with research methods, such as artificial intelligence (AI)–assisted acupoint selection and functional magnetic resonance imaging (fMRI), new breakthroughs have been achieved in exploring the mechanisms of acupuncture in treating diabetic complications [11–15]. This review systematically summarizes the types and pathogenesis of macrovascular and microvascular complications of diabetes, compiles evidence on the efficacy of acupuncture in treating these two types of complications, analyzes the potential mechanisms of action of acupuncture, and integrates current research hotspots in modern technology to provide a reference for the innovative application of acupuncture in the treatment of diabetic complications.

## 2. Macrovascular Complications

**Figure 1:**
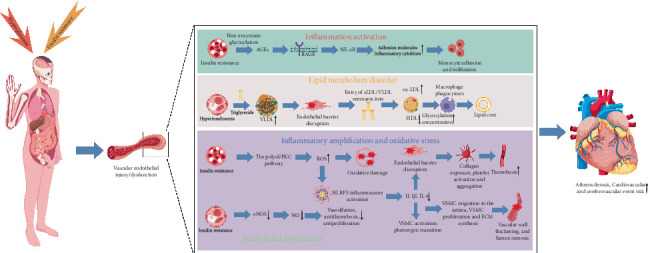
Pathogenesis of diabetic macrovascular complications. The cause of diabetic macrovascular complications is the accelerated atherosclerosis process under insulin dysregulation, which is initiated by vascular endothelial injury that triggers a cascade of multidimensional pathological networks. Hyperglycemia activates NF-*κ*B via the advanced glycation end product–receptor for AGEs (AGE–RAGE) axis, inducing inflammatory factor expression. Insulin resistance impairs eNOS activity, disrupting the endothelial barrier. Lipid metabolic disorder leads to ox-LDL formation, which activates NLRP3 inflammasomes. Hyperglycemia induces mitochondrial ROS production via polyol pathways, reinforcing inflammatory responses. Ultimately, VSMCs transform and proliferate, resulting in a thickening of vessel walls and narrowing of lumens. Further, platelet activation and aggregation accelerate thrombosis, causing atherosclerosis in diabetes characterized by earlier onset, diffuse lesions, and vulnerable plaques.

Approximately 40% of individuals with diabetes worldwide die from cardiovascular diseases, and the risk of cardiovascular diseases in Chinese diabetic patients is two to four times higher than that in the general population [16]. The essence of diabetic macrovascular complications is an accelerated atherosclerotic process driven by multiple mechanisms under abnormal insulin states. The core of macrovascular complications lies in vascular endothelial injury as the initial hub, triggering a cascade of pathological networks involving lipid metabolism, inflammation, oxidative stress, and other factors [17, 18]. Hyperglycemia generates advanced glycation end products (AGEs) through nonenzymatic glycosylation. Binding of AGEs to the receptor for AGEs (RAGE) on endothelial cells activates the nuclear factor kappa-light-chain-enhancer of activated B cells (NF-*κ*B) pathway, which induces the expression of adhesion molecules and inflammatory factors, promoting monocyte adhesion and infiltration. Moreover, insulin resistance impairs the activity of endothelial nitric oxide synthase (eNOS), which reduces nitric oxide (NO) production and diminishes vascular vasodilation, antithrombotic functions, and antiproliferative functions, thus disrupting endothelial barrier integrity. Hyperinsulinemia promotes the liver to synthesize very low-density lipoprotein (VLDL) rich in triglycerides. Its remnants and small low-density lipoprotein (sLDL) easily penetrate the endothelial space, and they are oxidized into oxidized low-density lipoprotein (ox-LDL), which triggers macrophage phagocytosis to form lipid cores. The antiatherosclerotic function of high-density lipoprotein cholesterol (HDL-C) is weakened by glycosylation modification and reduced concentration, exacerbating lipid deposition. ox-LDL and AGEs activate the NOD-like receptor Protein 3 (NLRP3) inflammasome in macrophages and vascular smooth muscle cells (VSMCs), releasing proinflammatory cytokines, such as interleukin-1 beta (IL-1*β*) and interleukin-6 (IL-6). Moreover, hyperglycemia induces excessive production of mitochondrial reactive oxygen species (ROS) through several pathways, including the polyol and protein kinase C (PKC) pathways, which oxidatively damages vascular wall components and reinforces NF-*κ*B signaling, further amplifying endothelial injury and inflammatory responses. Inflammatory factors stimulate VSMCs to transform from a contractile phenotype to a synthetic phenotype, after which they migrate to the intima, proliferate, and synthesize extracellular matrix components, such as collagen. These activities lead to thickening of the vessel wall and luminal stenosis. Owing to exposed endothelial collagen and combined with fibrinolytic system imbalance, platelets are activated and aggregate, thus accelerating thrombus formation. These interconnected mechanisms result in atherosclerosis in diabetic states characterized by earlier onset, more diffuse lesions, and increased plaque vulnerability to rupture, ultimately triggering cardiovascular and cerebrovascular events. As one of the most disabling and life-threatening chronic complications of diabetes, diabetic macrovascular complications have garnered extensive attention regarding their clinical features, therapeutic challenges, and the potential value of acupuncture intervention [19] (Figure 1).

### 2.1. Coronary Heart Disease (CHD)

When cardiovascular involvement occurs, typical manifestations include stable angina and acute coronary syndrome. In severe cases, cardiovascular involvement can lead to cardiogenic shock or sudden cardiac death [20].

#### 2.1.1. Limitations of Current CHD Treatments

The current treatment system faces multiple challenges in addressing diabetic macrovascular complications. First, there is a significant contradiction in blood glucose control strategies. Although intensive hypoglycemic therapy can reduce the risk of microvascular complications, it may increase the incidence of severe hypoglycemia, and the improvement effect on macrovascular events is delayed due to the “metabolic memory” effect [21]. Second, in lipid-lowering therapy, approximately 20% of patients cannot tolerate the side effects of statins, such as myalgia and elevated liver enzymes, making it difficult to control low-density lipoprotein cholesterol (LDL-C) within the effective range. The long-term safety of novel proprotein convertase subtilisin/kexin Type 9 (PCSK9) inhibitors still requires further clinical observation [22, 23]. In addition, revascularization techniques have indication limitations. Only 40% of patients with multivessel coronary artery disease are suitable for percutaneous coronary intervention (PCI), while the surgical risk of coronary artery bypass grafting (CABG) significantly increases in high-risk patients with left ventricular ejection fraction < 30% [24]. Although antiplatelet therapy reduces the risk of in-stent thrombosis, it brings the hidden danger of bleeding. Dual antiplatelet therapy (DAPT) increases the risk of gastrointestinal bleeding [25]. These limitations collectively highlight the insufficiency of existing treatments in individualized application and safety balance, urgently calling for more synergistic therapeutic regimens.

#### 2.1.2. Mechanism of Acupuncture in the Treatment of CHD

In recent years, high-quality clinical studies have continuously confirmed the synergistic value of acupuncture in the treatment of diabetic macrovascular complications. In a previous study involving 134 patients with Type 2 diabetes mellitus complicated with CHD and angina pectoris, the patients were randomly divided into an acupuncture plus medication group and a medication group; both groups received conventional Western medicine treatment, and the acupuncture plus medication group received acupuncture treatment in addition to the Western medicine treatment. The results showed that after 8 weeks of treatment, the total effective rate of the acupuncture plus medication group was 93.8%, which was significantly higher than 79.4% of the medication group. Moreover, the acupuncture plus medication treatment group exhibited better improvements in glucose metabolism, lipid metabolism, islet *β*-cell function, and cardiac function, indicating that the combined treatment controls the condition more effectively with a significant curative effect [26]. Experimental studies have further revealed that the efficacy of acupuncture in diabetic macrovascular complications is closely related to the regulation of the neuroendocrine–immune network. At the neuroregulatory level, fMRI has shown that acupuncture at Neiguan (PC6) inhibits the excessive activation of the insular cortex pain center and also enhances the prefrontal cortex regulatory ability of the prefrontal cortex over the autonomic nervous system, increasing the root mean square of successive differences (RMSSD) in heart rate variability (HRV)—a marker reflecting parasympathetic nerve tension. This suggests a shift in cardiac autonomic balance toward protective regulation [27]. In terms of metabolic regulation, acupuncture effectively improves insulin resistance by promoting GLP-1 hormone secretion and significantly reducing Angiotensin II (AngII) levels to inhibit abnormal proliferation of VSMCs, thus alleviating vascular remodeling pressure at its source [28, 29]. In terms of anti-inflammatory effects, acupuncture specifically inhibits the excessive activation of the NF-*κ*B signaling pathway, reducing levels of proinflammatory cytokines, such as IL-6 and tumor necrosis factor-alpha (TNF-*α*), while simultaneously upregulating the expression of anti-inflammatory cytokines, such as interleukin-10 (IL-10), forming a bidirectional immunomodulatory effect [30, 31]. In vascular protection mechanisms, acupuncture promotes phosphorylation of eNOS by activating the AMP-activated protein kinase (AMPK) pathway, which increases the production of the NO vasodilator. Acupuncture also inhibits ox-LDL-induced endothelial cell apoptosis, which reduces the apoptosis rate and maintains vascular endothelial integrity and functional activity [32, 33]. These studies confirm the positive intervention effects of acupuncture on diabetic macrovascular complications from different pathological sites, and the synergistic effect of acupuncture with conventional therapy is gradually gaining support from evidence-based medicine.

### 2.2. Ischemic Stroke (IS)

IS is the predominant cerebrovascular disease, and it typically presents with sudden unilateral limb weakness, speech impairment, and hemisensory disturbance at onset. Approximately 30% of patients with IS are left with varying degrees of neurological deficits [34].

#### 2.2.1. Limitations of Current IS Treatments

The current treatments for IS as a diabetic complication include intravenous thrombolysis, endovascular thrombectomy, and antiplatelet therapy, but these treatments have numerous limitations due to the pathophysiological characteristics of diabetic patients [35]. Atypical symptoms often lead to delayed medical consultation and missed therapeutic time windows. Severe vascular lesions result in low recanalization rates and high risks of reperfusion injury. Additionally, hyperglycemia increases the risk of hemorrhagic transformation, interferes with neurorepair, and creates contradictions in blood glucose management. Antiplatelet therapy is prone to drug resistance and elevated bleeding risk. Widespread atherosclerosis and insufficient collateral circulation also contribute to poor prognosis. Neuroprotective therapy has limited efficacy due to blood–brain barrier (BBB) disruption and the superimposition of multiple pathological mechanisms [36]. Poststroke rehabilitation is difficult due to peripheral vascular and neurological lesions. Traditional risk factor management faces challenges in glycemic control targets and drug–drug interactions in polypharmacy. Therefore, the overall treatment of IS requires exploring individualized strategies targeting diabetes-specific pathological links.

#### 2.2.2. Mechanism of Acupuncture in the Treatment of IS

**Figure 2:**
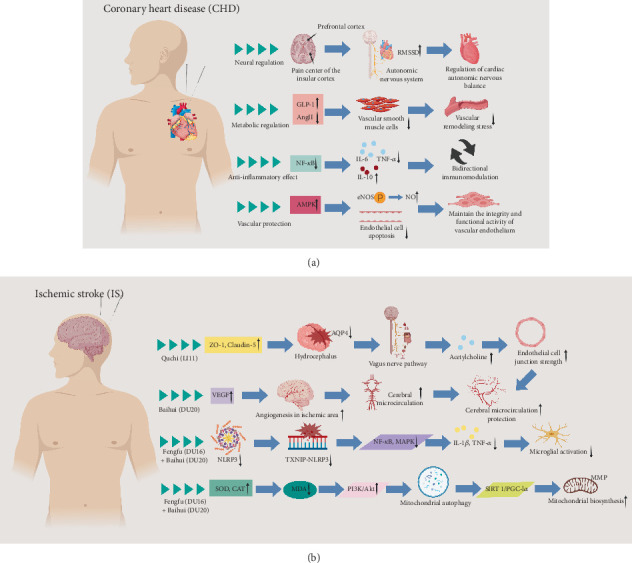
Possible mechanisms of acupuncture treatment for CHD and IS. (a) The efficacy of acupuncture in diabetic macrovascular complications is associated with the regulation of the neuroendocrine–immune network. Neuroregulation enhances autonomic nervous control, and metabolic regulation promotes GLP-1 secretion and reduces AngII. Moreover, anti-inflammatory effects inhibit the NF-*κ*B pathway, and vascular protection activates the AMPK pathway, increases NO production, and suppresses endothelial cell apoptosis. (b) Acupuncture at Quchi (LI11) upregulates ZO-1 and claudin-5 to inhibit brain edema, and it activates the vagus nerve to strengthen endothelial junctions. Acupuncture at Quchi (LI11) combined with Baihui (GV20) stimulates VEGF for neovascularization and microcirculation improvement. EA at Baihui (GV20) and Fengfu (GV16) suppresses NLRP3 inflammasome activation, suppresses NF-*κ*B/MAPK pathways, enhances antioxidant enzyme activity, and activates PI3K/Akt signaling to regulate mitochondrial function. Thus, different acupoints provide targeted evidence for clinical acupoint selection and protocol optimization of acupuncture in the treatment of IS by precisely regulating neuroprotective and inflammatory pathways.

Studies have shown that for diabetic patients taking antidiabetic medications, receiving at least three acupuncture treatments combined with conventional drug therapy reduces stroke-related mortality compared with conventional drug therapy alone. A secondary analysis after propensity score matching (PSM) has revealed that the risk of stroke-related death in an acupuncture group is significantly lower than that in a nonacupuncture group, suggesting that acupuncture effectively reduces the risk of stroke-related death in diabetic patients [37]. Additionally, studies have indicated that EA specifically enhances physiological activities related to blood glucose metabolism regulation, neuronal structure protection, and motor function repair in patients with diabetic stroke, and EA also improves patients' quality of life scores [38]. Basic research has shown that acupuncture at Quchi (LI11) upregulates the expression of tight junction proteins (zona occludens-1 [ZO-1] and claudin-5), inhibits Aquaporin 4 (AQP4)–mediated brain edema, and activates the vagus nerve pathway to release acetylcholine, enhancing endothelial cell junctions. Stimulation at Baihui (GV20) promotes vascular endothelial growth factor (VEGF) expression, induces angiogenesis in the ischemic area, and improves cerebral microcirculation [39, 40]. EA at Baihui (GV20) and Fengfu (GV16) inhibits the activation of the NLRP3 inflammasome, blocks the binding of thioredoxin-interacting protein (TXNIP) to NLRP3, and reduces the release of proinflammatory cytokines (such as IL-1*β* and TNF-*α*) by inhibiting the NF-*κ*B and mitogen-activated protein kinase (MAPK) pathways, thereby alleviating excessive activation of microglia [41]. EA at Baihui (GV20) and Fengfu (GV16) enhances the activity of antioxidant enzymes (such as superoxide dismutase [SOD] and catalase [CAT]), reduces malondialdehyde (MDA) content, promotes mitochondrial autophagy by activating the phosphatidylinositol 3-kinase (PI3K)/protein kinase B (Akt) pathway, and regulates the silent information Regulator 1 (SIRT1)/peroxisome proliferator–activated receptor gamma Coactivator 1 alpha (PGC-1*α*) pathway to enhance mitochondrial biogenesis and stabilize membrane potential [42]. These studies indicate that different acupoints provide targeted evidence for clinical acupoint selection and protocol optimization of acupuncture in the treatment of IS by precisely regulating neuroprotective and inflammatory pathways (Figure 2).

These multilevel mechanisms work synergistically to construct the scientific basis for acupuncture in improving diabetic macrovascular complications, demonstrating the profound convergence of traditional therapy and modern molecular biology. Current evidence indicates that acupuncture is an effective adjuvant therapy for diabetic macrovascular complications, with advantages of multitarget regulation, low side effects, and good tolerance. By regulating the neuroendocrine–immune network, acupuncture alleviates angina and accelerates cerebral blood reperfusion, and it has no risks of hypoglycemia, hepatorenal damage, or other complications associated with conventional therapies, thus providing a new strategy for the management of diabetic macrovascular complications.

## 3. Microvascular Complications

**Figure 3:**
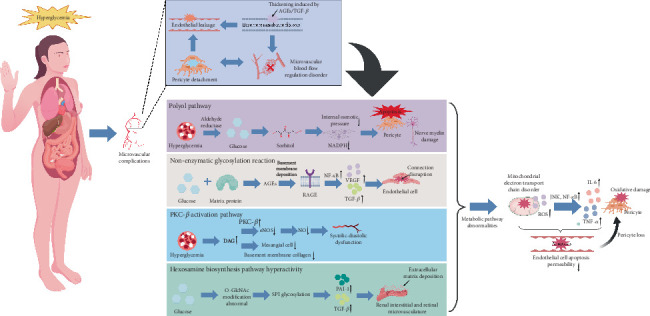
Pathogenesis of diabetic microvascular complications. The core of diabetic microvascular complications is a damaged network of the microvascular endothelial-pericyte-basement membrane axis formed by hyperglycemia-induced abnormalities in metabolic pathways (polyol, AGE–RAGE, PKC-*β*, and hexosamine) interacting with oxidative stress and inflammation. Metabolic disorders induce excessive mitochondrial ROS production, activating NF-*κ*B and other pathways, leading to disruption of endothelial tight junctions, pericyte apoptosis, and basement membrane thickening via AGE deposition and TGF-*β* stimulation. These actions form a vicious cycle of endothelial leakage-pericyte loss-basement membrane fibrosis, triggering glomerulosclerosis in kidneys, neovascularization in retinas, and neurotrophic disorders in nerves—highlighting the vessel-cell-matrix network injury characteristics.

The core of diabetic microvascular complications lies in the crosstalk among hyperglycemia-induced multiple metabolic pathway abnormalities, oxidative stress, and inflammatory responses, forming a damage network centered on the microvascular endothelial-pericyte-basement membrane axis. In the polyol pathway, aldose reductase converts excessive glucose into sorbitol, which leads to intracellular osmotic imbalance and depletion of reduced coenzymes, ultimately triggering pericyte apoptosis and nerve myelin injury. Nonenzymatic glycosylation reactions bind glucose to vascular matrix proteins to generate AGEs, which directly deposit in the basement membrane and activate NF-*κ*B via binding to RAGE, inducing the release of proinflammatory factors, such as VEGF and transforming growth factor beta (TGF-*β*), to exacerbate disruption of endothelial tight junctions [43]. Moreover, hyperglycemia activates protein kinase C-beta (PKC-*β*) via diacylglycerol (DAG), which inhibits eNOS, reduces NO production, and causes vascular dysfunction, but it promotes mesangial cell proliferation and basement membrane collagen synthesis. Hyperactivity of the hexosamine pathway leads to abnormal O-GlcNAc modification, which causes excessive glycosylation of transcription factor specificity Protein 1 (SP1) and drives the expression of certain genes, such as plasminogen activator inhibitor-1 (PAI-1) and TGF-*β*, thus accelerating extracellular matrix deposition in the renal interstitium and retinal microvessels. These metabolic pathway abnormalities collectively induce mitochondrial electron transport chain disorders, generating excessive ROS. ROS directly oxidize lipids in vascular endothelial and pericyte membranes, and they damage DNA structures and inhibit the activity of antioxidant enzymes, such as SOD [44]. Moreover, ROS activate c-Jun N-terminal kinase (JNK) and NF-*κ*B signaling pathways, prompting endothelial cells to release proinflammatory cytokines, such as IL-6 and TNF-*α*, which further damage pericytes and cause capillaries to lose support, ultimately creating microaneurysms. Endothelial cell apoptosis and tight junction disruption increase vascular permeability, leading to proteinuria and retinal edema. Pericyte loss impairs microvascular blood flow regulation, exacerbating local ischemia. The basement membrane thickens and stiffens due to AGE deposition and TGF-*β*-induced excessive matrix synthesis, thereby hindering substance exchange. These three elements interact to form a vicious cycle of endothelial leakage-pericyte detachment-basement membrane fibrosis, resulting in the following consequences: mesangial matrix expansion and glomerulosclerosis in the kidney, VEGF-dependent neovascularization induced by ischemia and hypoxia in the retina, and neurotrophic factor delivery disorders due to microvascular stenosis in peripheral nerves, aggravating axonal degeneration. This pathological process highlights the vascular-cell-matrix network damage characteristic of diabetic microangiopathy, which involves both universal and organ-specific mechanisms due to differences in microvascular structures. Early intervention to block metabolic pathway abnormalities and anti-inflammatory/antioxidant therapy have become critical for delaying complications [45]. As the most characteristic chronic damage due to diabetes, diabetic microvascular complications are pathologically based on the thickening of small vessel basement membranes, endothelial dysfunction, and microcirculatory disorders, and they primarily involve the retina, kidney, and peripheral nerves. The clinical complexity and therapeutic challenges of microvascular complications, as well as the potential value of acupuncture intervention, are emerging as hotspots in medical research (Figure 3).

### 3.1. Diabetic Retinopathy (DR)

DR is a classic example of diabetic microvascular complications. In the early stage, DR presents as blurred vision and floaters. As the disease progresses, microvascular leakage in the retina triggers macular edema, leading to a sharp decline in central vision [46]. When neovascularization ruptures, sudden vitreous hemorrhage also causes abrupt vision loss or even blindness [47].

#### 3.1.1. Limitations of Current DR Treatments

The core pathology of DR includes vasculopathy and neuropathy, with the two processes mutually promoting each other. Traditional treatments only target vascular abnormalities, lacking effective intervention for early neural damage. Hyperglycemia-induced oxidative stress, inflammatory factors, and metabolic products continuously damage nerve cells, and existing approaches fail to block this process, leading to reduced contrast sensitivity, color vision abnormalities, and other early visual function impairments in some patients, even without obvious vascular lesions. Laser photocoagulation for retinopathy seals leaking blood vessels but may damage surrounding normal retinal tissue, causing visual field defects [48]. Intravitreal injection of anti-VEGF agents rapidly resolves macular edema; however, frequent injections are required, and the risks of ocular infection and the economic burden of long-term treatment cannot be ignored [49].

#### 3.1.2. Mechanism of Acupuncture in the Treatment of DR

In DR, acupuncture combined with conventional therapy significantly improves the best corrected visual acuity (BCVA) in patients. After 12 weeks of treatment, visual acuity chart readings show a notable increase, and fundus fluorescein angiography reveals a reduction in the retinal microvascular leakage area; in addition, the central macular thickness (CMT) decreases after 12 weeks of treatment [50]. In terms of microcirculatory regulation, acupuncture dilates retinal and renal microvessels, increasing local blood flow. Laser Doppler flowmetry shows that acupuncture enhances retinal microcirculatory blood flow velocity and increases renal cortical blood flow, which is closely related to its promotion of NO production and inhibition of endothelin-1 (ET-1) release [32, 51]. Anti-inflammatory activity and antioxidative stress represent another core mechanism. Acupuncture inhibits the NF-*κ*B signaling pathway, which reduces proinflammatory factors (such as TNF-*α* and IL-6) in retinal and renal tissues but upregulates the activity of antioxidant enzymes, such as SOD and glutathione peroxidase (GSH-Px), to alleviate oxidative damage to the vascular endothelium [30, 33].

### 3.2. Diabetic Nephropathy (DN)

Proteinuria is an early sign of DN, and microalbuminuria marks the initiation of renal injury. Subsequently, urinary protein gradually increases, accompanied by elevated serum creatinine, edema, and hypertension, eventually progressing to end-stage renal disease [52, 53].

#### 3.2.1. Limitations of Current DN Treatments

With regard to etiologic treatment, although strict glycemic control slows the progression of diabetic microvascular complications, it is difficult to reverse existing vascular damage. Moreover, intensive hypoglycemic regimens may increase the risk of hypoglycemia, particularly limiting the safety of medication use in elderly patients and those with renal insufficiency [54]. Regarding blood pressure control, while angiotensin-converting enzyme inhibitor (ACEI)/Angiotensin II receptor blocker (ARB) drugs reduce proteinuria, approximately 15%–20% of patients cannot tolerate them due to side effects, such as dry cough and hyperkalemia. Additionally, the efficacy of these drugs significantly declines in patients who have already entered the stage of renal insufficiency [55].

#### 3.2.2. Mechanism of Acupuncture in the Treatment of DN

For DN, EA stimulation at certain acupoints, such as Sanyinjiao (SP6), Shenshu (BL23), and Guanyuan (CV4), reduces 24-h urinary protein quantification and serum creatinine levels, and it improves several symptoms, such as fatigue and edema, in patients. The efficacy of EA stimulation is comparable to ARB drugs but without the risk of hyperkalemia [56]. Additionally, acupuncture reduces proteinuria by regulating the expression of podocyte-related proteins in DN. Studies have demonstrated that acupuncture increases the protein expression of nephrin in renal tissue and decreases the incidence of foot process fusion, protecting the glomerular filtration barrier at the structural level [12]. Studies have also indicated that EA treatment reduces serum IL-1*β* and IL-6 levels in DN mice, exerting protective effects against renal injury. EA inhibits the high-mobility group Box 1 (HMGB1)/NLRP3/NF-*κ*B pathway, and HMGB1 inhibition enhances the anti-inflammatory effect of EA on DN by suppressing the NLRP3/NF-*κ*B pathway [57]. Combined acupuncture and moxibustion alleviates oxidative stress and renal injury in contrast-induced nephropathy (CIN) diabetic rats, possibly by downregulating the expression of renal Fas and FasL genes and proteins, with acupuncture showing a synergistic effect with moxibustion [58].

### 3.3. DPN

DPN is characterized by symmetrical distal sensory abnormalities, with patients often reporting numbness, tingling, or burning sensations in the distal extremities, presenting in a glove-and-stocking distribution. In severe cases, hyperalgesia may occur, and diabetic foot ulcers can even develop due to loss of sensation in the feet [59].

#### 3.3.1. Limitations of Current DPN Treatments

Controlling blood glucose as basic therapy can delay the progression of early lesions, but it cannot reverse established nerve damage. Even in some patients with long-term glycemic control, neuropathy continues to deteriorate due to multiple mechanisms, such as oxidative stress, inflammatory responses, and polyol pathway activation. Neurotrophic drugs exert effects by supplementing methylcobalamin or providing antioxidant activity, but their clinical efficacy is limited. Methylcobalamin promotes axonal regeneration only in mild nerve injury, with minimal effects on severe demyelination or axonal degeneration [60]. The antioxidant effect of alpha-lipoic acid is constrained by its short metabolic half-life and insufficient tissue penetrability, and the conclusions regarding the pain-relieving efficacy of alpha-lipoic are divergent [61]. Traditional treatments for DPN remain at the level of symptomatic therapy; they lack blockade of upstream mechanisms of nerve injury and multipathway collaborative intervention, and they fail to achieve individualized precision medicine. This leads to poor pain control in some patients, who gradually progress to sensory loss and increased ulcer risk, becoming important predisposing factors for severe complications, such as diabetic foot.

#### 3.3.2. Mechanism of Acupuncture in the Treatment of DPN

In the treatment of neuropathy, warm acupuncture–moxibustion at Zusanli (ST36), Yanglingquan (GB34), and Taichong (LR3) for 8 weeks reduces the visual analog scale (VAS) score of pain, increases motor nerve conduction velocity (MCV), and significantly improves hyperalgesia compared with pretreatment, and the combined treatment is superior to the pure drug treatment [62]. In terms of neuroprotection, acupuncture promotes the expression of nerve growth factor (NGF) and brain-derived neurotrophic factor (BDNF), and it repairs damaged Schwann cells. Electron microscopy observation has revealed that the myelin sheath thickness of nerve fibers increases compared with the model group, and the integrity of axonal structure is significantly improved [59, 63]. Basic research has shown that EA ameliorates neuropathic hyperalgesia, reduces proinflammatory cytokines, decreases the production of AGEs, decreases the levels of RAGE, and regulates the glyoxalase (GLO) system. The mechanism of EA may be related to metabolic regulation and secondary effects on the GLO–AGE–RAGE axis [64]. In the spinal cord of DPN rats, acupuncture downregulates the expression of P2X purinergic Receptor 4 (P2X4) and OX42, as well as reduces the levels of the C-X-C chemokine Receptor 3 (CXCR3), TNF-*α*, IL-1*β*, and IL-6 inflammatory factors, and acupuncture also decreases the levels of glycosylated serum protein (GSP) and lipid metabolism, thereby alleviating DPN in rats [65].

### 3.4. Diabetic Autonomic Neuropathy (DAN)

DAN is caused mainly by damage to the autonomic nervous system due to long-term hyperglycemia. DAN involves multiple systems, including the cardiovascular, digestive, urogenital, and respiratory systems, seriously impairing patients' quality of life and prognosis [66].

#### 3.4.1. Limitations of Current DAN Treatments

The traditional treatment of DAN is based on strict glycemic control; however, hyperglycemia is not the sole driver of the lesion. Synergistic mechanisms, such as oxidative stress, accumulation of AGEs, and microvascular ischemia, independently damage autonomic nerves. Even with optimal glycemic control, neuropathy may still progress in some patients, and it is difficult to completely halt the disease course through glycemic control alone. Secondly, *α*-receptor agonists used for orthostatic hypotension may cause side effects, such as palpitations and hypertension, with long-term efficacy waning [67]. Gastrointestinal prokinetics commonly used in patients with gastroparesis only temporarily improve delayed emptying, fail to reverse structural damage to gastrointestinal nerves, and have limited efficacy in severe cases. Moreover, PDE5 inhibitors for erectile dysfunction are often ineffective in patients with severe degeneration of nerve fibers [68, 69]. Furthermore, nerve repair approaches are lacking. Although traditional neurotrophic agents are widely used, their clinical benefits show significant individual variability, with weak efficacy in patients with moderate to severe lesions. Additionally, there is a lack of large-scale evidence-based medical evidence supporting clear mechanisms of neurotrophic agents for promoting axonal regeneration and myelin repair. For patients with irreversible nerve fiber disruption, existing drugs do not achieve functional reconstruction [70]. In addition, when the lesion simultaneously involves multiple systems, such as the cardiovascular, digestive, and urinary systems, traditional treatment requires the combination of multiple symptomatic drugs, which increases the risk of drug interactions and may reduce patient compliance due to complex medication regimens, making it difficult to achieve synergistic improvement of multisystem lesions [71].

#### 3.4.2. Mechanism of Acupuncture in the Treatment of DPN

**Figure 4:**
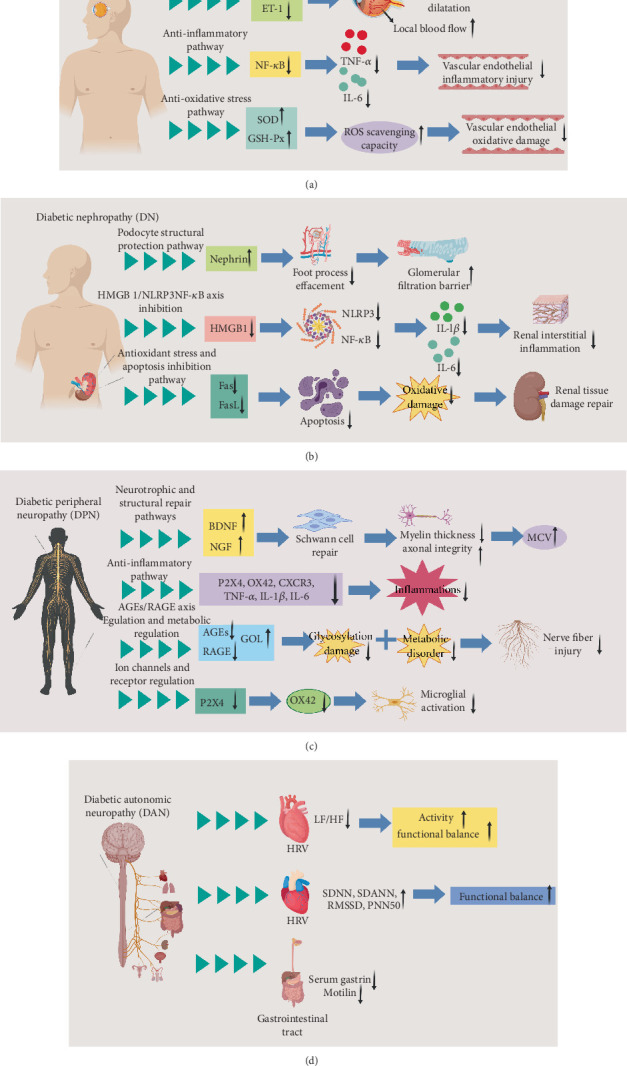
Possible mechanisms of acupuncture for DR, DN, and DPN. (a) Acupuncture combined with conventional therapy improves BCVA in DR patients, reduces retinal microvascular leakage, and decreases CMT. Acupuncture dilates retinal and renal microvessels to increase blood flow by promoting NO production, inhibiting ET-1, suppressing the NF-*κ*B pathway to reduce proinflammatory factors (TNF-*α* and IL-6), and upregulating antioxidant enzymes (SOD and GSH-Px). (b) EA for DN reduces 24-h urine protein and serum creatinine, and it alleviates symptoms comparable to the level of ARB drugs without hyperkalemia risk. EA protects the glomerular filtration barrier by regulating podocyte proteins, inhibiting the HMGB1/NLRP3/NF-*κ*B pathway to promote anti-inflammatory effects, downregulating Fas/FasL to mitigate oxidative stress, and synergizing with moxibustion. (c) Warm acupuncture at Zusanli (ST36) increases nerve conduction velocity and relieves allodynia in DPN, and it promotes the expression of NGF and BDNF to repair Schwann cells and improve myelin/axon structure. In addition to reducing proinflammatory factors and AGEs/RAGE, EA regulates the glyoxalase system and downregulates P2X4, OX42, and inflammatory cytokines for neuropathy relief. (d) Acupuncture treatment has an adjunctive effect on cardiovascular autonomic neuropathy, which can improve HRV and autonomic nerve regulatory function. Medium-frequency pulse stimulation at Tianshu (ST25) acupoints can alleviate diabetic gastrointestinal autonomic neuropathy, relieve symptoms, and regulate hormones.

In terms of autonomic nervous function regulation, acupuncture intervention for cardiovascular autonomic neuropathy has shown clear effects. Acupuncture at the Lingtai (GV10) and Shendao (GV11) acupoints increases both the low-frequency (LF) and high-frequency (HF) values of HRV but decreases the LF/HF ratio. This directly reflects enhanced activity and improved functional balance of the sympathetic and vagus nerves, accompanied by a reduced frequency of resting tachycardia attacks [72]. Additionally, clinical studies have shown that interventions combining lipoic acid with acupuncture at the Neiguan (PC6), Pishu (BL20), Zusanli (ST36), and Taixi (KI3) acupoints achieve positive progress; after 2 weeks of treatment, the total effective rate significantly increases, and HRV indicators, such as SDNN, SDANN, RMSSD, and PNN50, are significantly improved. Holter electrocardiogram analysis further revealed enhanced autonomic regulatory function. These indicators collectively suggest that lipoic acid combined with acupuncture may protect against diabetes-related cardiovascular autonomic nerve damage by improving neuro-oxidative stress and regulating the balance between sympathetic and vagal tone [73]. In patients with diabetic gastrointestinal autonomic neuropathy who receive medium-frequency pulse electrical stimulation at bilateral Tianshu (ST25) acupoints, the gastrointestinal symptom scores, serum gastrin levels, and serum motilin levels are markedly decreased, and various symptoms, such as anorexia, abdominal distension, and early satiety, are effectively relieved. These findings indicate that medium-frequency pulse electrical stimulation improves gastrointestinal autonomic nerve function and regulates gastrointestinal hormones [74] (Figure 4).

In summary, diabetic microvascular complications, characterized by their insidiousness, progressiveness, and multisystem damage, pose a challenge in diabetes management. Although traditional therapies have a certain role in delaying disease progression, they lack effective interventions for established organ damage. Acupuncture, by virtue of its advantages in multitarget regulation, has potential in improving clinical symptoms, retarding lesion progression, and protecting target organs. The mechanisms of acupuncture involve multiple levels, including microcirculatory improvement, anti-inflammatory effects, antioxidative effects, neuroprotection, and structural repair.

## 4. Other Complications of Diabetes

The complications induced by diabetes extend beyond those summarized above, and they involve multiple systems. In the ocular system, hyperglycemia accelerates lens opacification, advancing the onset age of cataracts and increasing its incidence by two- to fivefold compared to the general population [75]. Skin and soft tissues are also frequently affected; hyperglycemia creates a favorable environment for bacterial and fungal growth, making infectious diseases, such as furuncles, carbuncles, paronychia, tinea pedis, and candidiasis, more common [76]. Metabolic and acute complications should not be overlooked. When Type 1 diabetes or severe insulin deficiency occurs in Type 2 diabetes, diabetic ketoacidosis (DKA) easily emerges, characterized by hyperglycemia, ketosis, and acidosis, which can be life-threatening [77]. Most importantly, long-term illness impacts psychological and social health. Psychological issues, such as anxiety and depression, often accompany the disease, while the burden of blood glucose monitoring and medication may reduce patient compliance, forming a vicious cycle in disease management [78]. Acupuncture therapy, with its advantages of multitarget integrated regulation, low side effects, rapid efficacy, and sustained efficacy, compensates for the limitations of traditional therapies in managing the ever-emerging diabetic complications. For ocular complications, studies have reported that acupuncture at the Jingming (BL2), Chengqi (ST1), and Cuanzhu (BL2) acupoints improves ocular microcirculation, slowing the progression of DR and improving vision in some patients [79]. For skin and soft tissue infections, acupuncture dilates capillaries, enhances local blood flow, and promotes wound healing. Moreover, the anti-inflammatory effect of acupuncture reduces the incidence of infections, such as furuncles and tinea pedis; combined with external Chinese medicine, acupuncture significantly shortens the course of disease [80]. In adjuvant treatment of metabolic emergencies, acupuncture at the Shuigou (GV26), Chengjiang (CV24), Jinjin (EX-HN12), and Yuye (EX-HN13) acupoints relieves dehydration and acidosis in DKA patients, accelerating consciousness recovery when combined with Western emergency measures [81]. For psychological issues induced by long-term illness, acupuncture at the Baihui (GV20), Sishencong (EX-HN1), and Taixi (KI3) acupoints regulates the neuroendocrine system, promotes endorphin release, and improves anxiety/depression symptoms. Acupuncture also enhances functional coupling of the prefrontal–hippocampal circuit to improve treatment compliance [82]. Additionally, EA reduces proinflammatory factors by inhibiting the NF-*κ*B pathway, enhances the activity of antioxidant enzymes (such as SOD and GSH-Px), and indirectly alleviates oxidative damage in skin infections and neuropathy [83] (Table 1).

Although acupuncture has demonstrated promising efficacy in treating diabetic complications, more high-quality clinical studies are needed to further explore its specific mechanisms and long-term effects. In conclusion, as a safe and effective therapeutic modality, acupuncture may provide new ideas and approaches for managing complications in diabetic patients.

## 5. The Integration and Innovative Application of Modern Technology in Acupuncture

With the development of precision medicine and translational medicine, acupuncture therapy is transitioning from empirical medicine to evidence-based medicine. The interdisciplinary integration of modern technology and acupuncture has provided innovative approaches for the prevention and treatment of diabetic complications. This section highlights current research progress, key mechanisms, and unresolved issues from two aspects, namely, precision acupuncture techniques and combined therapeutic strategies.

### 5.1. Precision Acupuncture Technology

The complexity of diabetic complications and individual patient variations has led to significant limitations in traditional empirical acupoint selection. The unclear association among genotypes, metabolic phenotypes, and acupoint sensitivity in patients with diabetic complications has resulted in acupoint tolerance in some individuals. Studies have shown that patients carrying the COMT Val/Val genotype have weaker responses to conventional EA stimulation, with only a 50% pain relief rate—significantly lower than those with Val/Met or Met/Met genotypes—indicating that the COMT Val158Met genotype may influence pain sensitivity to EA [84]. Additionally, the regulatory effects of acupuncture on complex endocrine pathways, such as the hypothalamic–pituitary–adrenal (HPA) axis, lack real-time monitoring methods, making it difficult to precisely assess treatment intensity [85]. Research has found that cortisol levels significantly fluctuate after acupuncture in diabetic patients with anxiety, but traditional methods cannot capture these dynamic changes [86]. The introduction of AI and fMRI technology has addressed these challenges. A syndrome-symptom-acupoint association model constructed based on the Apriori algorithm integrates traditional Chinese medicine (TCM) syndrome classification, clinical symptom data, and acupoint compatibility rules to achieve precise acupoint prescription recommendations. A multicenter study on patients with diabetic gastroparesis has reported that AI-generated syndrome-specific acupoint combination protocols shorten gastric emptying time and reduce dyspepsia symptom scores, significantly outperforming traditional empirical acupoint selection protocols [87]. Resting-state fMRI (rs-fMRI) has provided a visualization tool for mechanistic studies of acupuncture in the treatment of painful diabetic peripheral neuropathy (PDPN). rs-fMRI clarifies the negative correlation between acupuncture-mediated regulation of amplitude of LF fluctuations (ALFF) in pain-related brain regions and VAS pain scores, and it also adjusts acupuncture parameters based on dynamic changes in brain functional connectivity (FC) networks, improving the regulatory efficiency of central sensitization in PDPN patients [88]. However, many scientific questions remain to be solved. Diabetic complications are complex, and individual patient differences are significant, leading to numerous limitations in traditional empirical acupoint selection. The unclear association among patient genotypes, metabolic phenotypes, and acupoint sensitivity gives rise to acupoint tolerance, while the regulatory effects of acupuncture on complex endocrine pathways are difficult to assess precisely, affecting treatment efficacy. AI and rs-fMRI technologies have brought breakthroughs to acupuncture. AI enables precise acupoint prescription recommendations, and rs-fMRI assists in mechanistic research. Nevertheless, several challenges, such as insufficient adaptability of AI models to TCM theories and vague acupoint-specific mechanisms in brain functional imaging, still need further investigation.

### 5.2. Combined Therapeutic Strategies

In the field of combined therapeutic strategies, the limitations of single therapies have prompted the combined application of acupuncture with modern drugs and technologies. Although GLP-1 receptor agonists and SGLT2 inhibitors effectively control blood glucose, they have risks of gastrointestinal reactions or ketosis, and mesenchymal stem cell transplantation for diabetic foot faces challenges of low homing efficiency and cell viability [5, 89]. Clinical practice has indicated that in the treatment of DPN, combining acupuncture with medication for 4 weeks significantly reduces the Toronto Clinical Scoring System (TCSS) score and improves motor and sensory conduction velocities of the median and common peroneal nerves, outperforming monopharmaceutical therapy. This may occur via regulating serum lipid metabolism by significantly downregulating metabolites (such as lysophosphatidic acid [LPA] and lysophosphatidylcholine [LPC]), which interferes with glycerophospholipid metabolic pathways and ameliorates neuronal oxidative stress and demyelination [90]. In the field of diabetic skin wound healing, the combined therapy of adipose-derived stem cell (ADAS)–loaded cryogel and periwound acupuncture significantly promotes wound repair. This combined therapy enhances the wound closure rate in diabetic rats and accelerates granulation tissue formation, and it promotes abundant microvascular generation and intact epithelial regeneration. Mechanistically, acupuncture elevates local wound temperature, promotes vasodilation, promotes blood perfusion, activates the complement system, and activates chemokine pathways. In addition, the ability of biomaterials to absorb exudate and inhibit bacterial infection creates a suitable microenvironment for stem cell engraftment. ADAS-loaded biomaterials secrete certain growth factors, such as stromal cell–derived Factor 1 (SDF-1), to recruit endogenous stem cells and regulate macrophage polarization, with acupuncture further enhancing this immunomodulatory effect by reducing proinflammatory responses and promoting angiogenesis. Together, ADAS-loaded biomaterials and acupuncture accelerate inflammation resolution and tissue regeneration in chronic wounds by regulating the C3a/C5a-SDF-1/TGF-*β*1 signaling axis, offering a novel synergistic strategy for refractory diabetic wounds [91]. However, critical questions remain. The optimal timing for combining acupuncture with drugs is unknown, and the method for synergistically optimizing cryogel replacement intervals with acupuncture frequencies remains unclear. Further, the impact of different acupuncture intensities on the activation degree of the C3a/C5a complement system needs further clarification.

## 6. Discussion

Diabetes mellitus and its complications have become a major global public health challenge. Conventional Western medicine faces limitations in efficacy and safety risks in the management of complications, while acupuncture, with its advantages of multitarget regulation, few side effects, and synergistic effect, is gradually becoming an important part of the comprehensive treatment of diabetes. Compared with the limitations of single-target intervention in existing Western medicine treatments, this review integrates data from multiple randomized controlled trials and basic research experiments to suggest that acupuncture is superior to drug treatment alone in improving diabetic macrovascular and microvascular complications. Compared with similar systematic reviews [92], this review includes the latest evidence on the application of AI technology. By systematically sorting out the clinical evidence and potential mechanisms of acupuncture in diabetic macrovascular, microvascular, and other complications, combined with the innovative applications of modern technologies, this review provides theoretical support for the precision and scientific development of acupuncture.

Clinical evidence indicates that acupuncture significantly improves both diabetic macrovascular and microvascular complications. In macrovascular diseases, acupuncture relieves angina, improves cerebral blood flow, and improves lower limb circulation via mechanisms involving inhibition of the NF-*κ*B inflammatory pathway, enhancement of vascular endothelial function, and regulation of autonomic nervous balance. In microvascular complications, acupuncture delays retinopathy, reduces proteinuria, and repairs peripheral nerves by dilating microvessels, inducing anti-inflammatory effects, inducing antioxidative effects, and promoting the expression of the NGF and BDNF, as well as the activation of their downstream TrkA/TrkB receptors. Basic research has revealed that the efficacy of acupuncture originates from its multidimensional regulation of the metabolic-inflammatory-neurovascular network—from activating signaling pathways (via GLP-1 and SDF-1) at the molecular level to repairing endothelial cells and Schwann cells at the cellular level—ultimately achieving holistic improvement of organ function.

The introduction of technologies, such as AI and fMRI, has driven the transformation of acupuncture from empirical acupoint selection to precision-based practice. By integrating TCM syndrome data and clinical symptoms, the AI-based syndrome-symptom-acupoint correlation model provides individualized acupoint prescription schemes for complex conditions, such as diabetic gastroparesis, with a higher effective rate compared to traditional syndrome differentiation-based acupoint selection [93]. rs-fMRI technology visualizes the regulatory effects of acupuncture on pain-related brain regions. The discovered regulatory mechanism of the prefrontal–hippocampal circuit supplements modern evidence for the TCM theory of “meridians-brain fu” [94]. In combined therapies, the synergistic effect of moxibustion combined with silver ion–containing gel in accelerating the healing of diabetic foot ulcers [95] demonstrates the synergistic potential between traditional therapies and modern biomedicine. Despite the clear efficacy of acupuncture, its development still faces issues, such as fragmented mechanistic explanations, insufficient high-quality clinical evidence, and lack of technical standardization. According to the quality assessment of Cochrane systematic reviews of acupuncture by Ji et al., 94% of current clinical studies do not consider clear research designs as inclusion criteria, and 52% of studies fail to fully investigate and explain publication bias. Meanwhile, only 5.3% of randomized controlled trials cited previous systematic reviews when elaborating on the study rationale, indicating that the utilization rate of existing evidence in original studies is extremely low [96–98]. In mechanistic research, the explanation of the crosstalk in the neuroendocrine–immune network lags behind the understanding of multiorgan interaction mechanisms in diabetes in the field of Western medicine.

Breakthroughs can be made in future research on the basis of three aspects. First, future research should establish an international multicenter cohort for the combined treatment of acupuncture and SGLT2 inhibitors, obtain hard endpoint data (such as the incidence of cardiovascular events through long-term clinical follow-up), and fill the gap in long-term safety evidence regarding the combination with new-type hypoglycemic drugs [99]. Second, researchers should construct a database of interaction networks between TCM syndromes and multiomics, and they should use network pharmacology methods to analyze the molecular mapping of the theory of simultaneous regulation of the liver, spleen, and kidney in the neuroendocrine–immune network, breaking through the limitations of single-target research [100, 101]. Third, researchers should formulate AI-based industry standards for acupoint stimulation parameters, including current intensity, pulse frequency, and syndrome adaptation thresholds, to address technical standardization challenges. These directions can only achieve breakthroughs by integrating Chinese and Western medicine and multidisciplinary collaboration [102, 103].

## 7. Conclusion

Acupuncture, with its advantage of holistic regulation, compensates for the shortcomings of traditional therapies, and the integration of acupuncture with modern technologies accelerates its transition from an adjuvant to a precision therapy. Although in-depth research into the mechanism and additional evidence is still needed, the safety and potential cost-effectiveness of acupuncture have provided important supplements for the management of diabetic complications. With the expansion of integrative medicine research combining traditional Chinese and Western approaches, acupuncture is poised to play a more pivotal role in the comprehensive prevention and treatment of diabetes, offering innovative solutions to improve patients' quality of life and reduce healthcare burdens.

## Figures and Tables

**Table 1:** A summary of acupuncture points and mechanisms for diabetic complications.

**Type of complications**	**Disease**	**Evidence level**	**Acupoints**	**Mechanism**	**References**
Macrovascular complications	CHD	Clinical trial	Multiacupoint combined therapy	The total effective rate of the acupuncture plus medication group was significantly higher than that of the medication group, and it was superior in improving glucose and lipid metabolism, islet *β*-cell function, and cardiac function	[26]
		Clinical trial	Neiguan (PC6)	Inhibiting excessive activation of the insular cortex pain center also enhances prefrontal cortex regulation of the autonomic nervous system, increasing HRV's RMSSD and suggesting cardiac autonomic balance tilts toward protective regulation	[27]
		Animal experiment	Neiguan (PC6); Tongli (HT5); Huantiao (GB30); Yanglingquan (GB34); Xuanzhong (GB39); Zusanli (ST36); Sanyinjiao (SP6)	Promote the secretion of GLP-1, improve insulin resistance, downregulate the level of AngII, and inhibit the abnormal proliferation of vascular smooth muscle cells	[28, 29]
		Animal experiment	Quchi (LI11); Zusanli (ST36)	Inhibit the excessive activation of the NF-*κ*B signaling pathway, reduce the levels of proinflammatory factors such as IL-6 and TNF-*α*, and simultaneously upregulate the expression of anti-inflammatory factors like IL-10	[30, 31]
		Animal experiment	Neiguan (PC6)	Activate the AMPK pathway to promote eNOS phosphorylation, thereby increasing the production of the vasodilatory factor NO. Simultaneously, it inhibits ox-LDL-induced endothelial cell apoptosis, reducing the apoptosis rate	[32, 33]
	IS	Clinical trial	Multiacupoint combined therapy	Secondary analysis after PSM showed that the risk of stroke-related death in the acupuncture group was significantly lower than in the nonacupuncture group, suggesting acupuncture can effectively reduce such risk in diabetic patients	[37]
		Clinical trial	Multiacupoint combined therapy	Electroacupuncture can enhance physiological activities related to glucose metabolism regulation, neuronal structure protection, and motor function repair in patients with diabetic stroke and can improve their quality of life scores	[38]
		Animal experiment	Quchi (LI11); Baihui (DU20)	Upregulate the expression of ZO-1 and Claudin-5, inhibit AQP4-mediated brain edema, simultaneously activate the vagus nerve pathway to release acetylcholine, enhance endothelial cell junctions, promote VEGF expression, induce angiogenesis in ischemic areas, and improve cerebral microcirculation	[39, 40]
		Animal experiment	Baihui (DU20); Fengfu (DU16)	Inhibit the activation of NLRP3 inflammasome and block the binding of TXNIP to NLRP3. Simultaneously, reduce the release of proinflammatory factors such as IL-1*β* and TNF-*α* by inhibiting NF-*κ*B and MAPK pathways. Enhance the activity of antioxidant enzymes like SOD and CAT, and decrease MDA content. Promote mitophagy by activating the PI3K/Akt pathway, regulate the SIRT1/PGC-1*α* pathway to enhance mitochondrial biogenesis, and stabilize membrane potential	[41, 42]

Microvascular complications	DR	Clinical trial	Multiacupoint combined therapy	Fundus fluorescein angiography shows a reduction in the area of retinal microvascular leakage and a decrease in CMT	[50]
		Animal experiment	Fengchi (GB20); Fengfu (DU16); Baihui (DU20); Zusanli (ST36); Quchi (LI11)	Promote the production of NO, inhibit the release of ET-1, increase the blood flow velocity in retinal microcirculation, and enhance renal cortical blood flow	[32, 51]
		Animal experiment	Multiacupoint combined therapy	Inhibit the NF-*κ*B signaling pathway, reduce proinflammatory factors such as TNF-*α* and IL-6 in retinal and renal tissues, and simultaneously upregulate the activity of antioxidant enzymes like SOD and GSH-Px	[30, 33]
	DN	Clinical trial	Sanyinjiao (SP6); Shenshu (BL23); Guanyuan (CV4)	The 24-h urinary protein quantification is reduced, serum creatinine levels decrease, and meanwhile, symptoms such as fatigue and edema in patients are improved	[56]
		Animal experiment	Multiacupoint combined therapy	The expression level of nephrin protein is increased, and the incidence of foot process fusion is reduced	[12]
		Animal experiment	Shenshu (BL23); Yishu (BL27); Zusanli (ST36); Sanyinjiao (SP6)	Serum levels of IL-1*β* and IL-6 are decreased, and the HMGB1/NLRP3/NF-*κ*B pathway is inhibited	[57]
		Animal experiment	Sanyinjiao (SP6); Shenshu (BL23); Pishu (BL20)	Alleviates oxidative stress and renal injury in CIN diabetic rats, and the mechanism may be related to downregulating the gene and protein expression of renal Fas and FasL	[58]
	DPN	Clinical trial	Zusanli (ST36); Yanglingquan (GB34); Taichong (LR3)	The VAS is significantly reduced, MCV is increased compared with pretreatment, and there is a remarkable improvement in allodynia	[62]
		Animal experiment	Multiacupoint combined therapy	Promote the expression of NGF and BDNF, and repair damaged Schwann cells	[59, 63]
		Animal experiment	Tianshu (ST25)	Improves neuropathic allodynia, reduces proinflammatory cytokines, decreases the production of advanced glycation end products (AGEs) and their receptor (RAGE), and regulates the glyoxalase system. The mechanism may be related to metabolic regulation and secondary effects on the glyoxalase (GLO)/AGE/RAGE axis	[64]
		Animal experiment	Feishu (BL13); Pishu (BL20); Shenshu (BL23)	Downregulates the expression of P2X4 and OX42, reduces the expression of inflammatory cytokines CXCR3, TNF-*α*, IL-1*β*, and IL-6, and decreases GSP levels and lipid metabolism in the spinal cord of DPN (diabetic peripheral neuropathy) rats, thereby alleviating DPN	[65]
	DAN	Clinical trial	Lingtai (GV10); Shendao (GV11)	The LF and HF values of HRV both increased, while the LF/HF ratio decreased. This directly reflects enhanced activity of the sympathetic and vagus nerves along with improved functional balance, accompanied by a reduced frequency of resting tachycardia episodes	[72]
		Clinical trial	Neiguan (PC6); Pishu (BL20); Zusanli (ST36); Taixi (KI3)	SDNN, SDANN, RMSSD, and PNN50 were all significantly improved, and ambulatory electrocardiogram analysis further showed enhanced autonomic nervous regulation function	[73]
		Clinical trial	Tianshu (ST25)	Gastrointestinal symptom scores decreased significantly, serum gastrin and motilin levels dropped markedly, and symptoms such as anorexia, abdominal distension, and early satiety were effectively relieved	[74]

Other complications of diabetes	Ocular complications	Clinical trial	Jingming (BL1); Chengqi (ST1); Cuanzhu (BL2)	Improves ocular microcirculation, slows the progression of diabetic retinopathy in patients, and enhances vision in some cases	[79]
	Skin and soft tissue infections	Clinical trial	Bloodletting cupping therapy	Dilates capillaries and enhances local blood flow	[80]
	Adjunctive therapy for metabolic emergencies	Clinical trial	Shuigou (DU26); Chengjiang (CV24); Jinjin (EX-HN12); Yuye (EX-HN13)	Alleviates dehydration and acidosis in patients with diabetic ketoacidosis (DKA) and in conjunction with Western medical emergency measures, accelerates consciousness recovery	[81]
	Psychological problems	Clinical trial	Baihui (DU20); Sishencong (EX-HN1); Taixi (KI3)	Regulates the neuroendocrine system, promotes endorphin release, improves anxiety and depression symptoms, and simultaneously enhances the functional coupling of the prefrontal–hippocampal circuit	[82]

Abbreviations: CHD, coronary heart disease; DAN, diabetic autonomic neuropathy; DN, diabetic nephropathy; DPN, diabetic peripheral neuropathy; DR, diabetic retinopathy; IS, ischemic stroke.

## Data Availability

Data sharing is not applicable to this article as no datasets were generated or analyzed during the current study.
